# Influence of *Tithonia diversifolia* biochar on selected soil physicochemical properties, leaf nutrient concentrations and broccoli growth

**DOI:** 10.1038/s41598-025-91844-w

**Published:** 2025-03-08

**Authors:** Taiwo Michael Agbede

**Affiliations:** Department of Agronomy, Adekunle Ajasin University, P.M.B. 001, Akungba-Akoko, Ondo State Nigeria

**Keywords:** ***Tithonia diversifolia*** Biochar, Soil bulk density, Moisture content, Soil chemical properties, Leaf nutrient concentrations, Biochemistry, Biological techniques, Environmental sciences

## Abstract

A screen house experiment was conducted to evaluate the effects of ***Tithonia diversifolia*** biochar on selected soil physicochemical properties. The study included five treatments with ***Tithonia diversifolia*** biochar applied at rates of 0, 10, 20, 30, and 40 t ha^− 1^, arranged in a completely randomised design with three replicates. Surface soil (0–15 cm depth) from the Iwo soil series (sandy loam) was collected from the Teaching and Research Farm of Adekunle Ajasin University, Akungba-Akoko, Ondo State, Nigeria. Each 10 kg soil sample was thoroughly mixed with the respective biochar rate and maintained at field moisture capacity for four weeks in the screen house before sowing broccoli seeds. Soil samples were analyzed for particle size distribution, bulk density, porosity, moisture content, pH, organic carbon, total nitrogen, available phosphorus, exchangeable potassium, calcium, and magnesium. Agronomic parameters measured included broccoli height, number of leaves, leaf area, stem girth, and fresh weight of broccoli biomass. Leaf nutrient concentrations of broccoli were also determined. Data were subjected to analysis of variance, and significant means were separated using Duncan’s multiple range test at *p* = 0.05. Results indicated that biochar-amended plots significantly improved soil physicochemical properties compared to the control. Biochar application also significantly increased broccoli height, number of leaves, leaf area, stem girth, leaf nutrient concentrations, and fresh weight of broccoli biomass. The application of tithonia biochar at rates of 10, 20, 30, and 40 t ha^− 1^ increased the fresh weight of broccoli biomass by 13%, 38%, 26%, and 23%, respectively, compared to the control. The application rate of 20 t ha^− 1^ was found to be the most beneficial, enhancing leaf nutrient concentrations and growth parameters. This study recommends the use of ***Tithonia diversifolia*** biochar as a soil amendment to improve soil quality of sandy loam and enhance broccoli productivity and quality.

## Introduction

Soil degradation significantly affects land productivity, reducing the quality of life and food security worldwide^1^. Effective strategies are essential for promoting improved soil management policies to meet the food demands of a growing global population^2^. Naturally-derived soil amendments, which enhance soil structure and water and nutrient retention, are vital tools in soil management. This is especially important in Sub-Saharan Africa (SSA), where soils are fragile, structurally weak, and low in organic matter and nutrients, such as in southwest Nigeria^3^.

One such amendment, biochar, is known to boost carbon sequestration and improve soil physical and chemical properties^4–6^. Biochar is produced through the pyrolysis of organic waste materials, a process that heats organic biomass at low temperatures or in the absence of oxygen^7,8^. The characteristics of biochar can vary depending on the pyrolysis temperature and the type of feedstock used^4,9^. Biochar is resistant to microbial decomposition and can remain in soils for many years, enhancing carbon sequestration over the long term^10^. This suggests that biochar can improve agronomic productivity when used as a soil amendment^11^.

As a potential substitute for soil organic matter (SOM), biochar can maintain soil biological activity, facilitating nutrient cycling in soils with low organic carbon content. Its application can enhance soil fertility, crop yields, plant growth, microbial abundance, and immobilize various soil contaminants. The large surface area of biochar, influenced by the type of feedstock and pyrolysis conditions, helps reduce fertilizer leaching and supplies additional nutrients to crops^12,13^. Biochar addition to soil has also been shown to decrease the leaching of nutrients such as nitrogen, phosphorus, and potassium^14,15^, decrease the nitrogen leaching and volatilization as well as increase nutrient use efficiency (NUE)^16^.

Vegetable growers in the forest-savanna transition zone of southwest Nigeria face several challenges, including lower fertility status, unique soil characteristics, and increased soil acidity resulting from consistent crop cultivation practices and the use of mineral fertilizers^17^. While chemical fertilizers can enhance crop productivity and sustain short-term productivity in agro-ecosystems, studies revealed that their excessive and indiscriminate application can lead to adverse effects such as a decline in soil quality and fertility, reduction in soil organic matter (SOM), soil acidification, nutrient imbalances, negative impacts on enzymatic activity, and risks to the copiotrophic community^8,18^. These challenges pose significant threats to long-term sustainability^19,20^. Research by Igalavithana et al.^21^ and Alburquerque et al.^22^ had shown that biochar can be beneficial as a soil amendment, improving the quality of agricultural soils. According to Sohi^23^, the biochar feedstock and production conditions can influence its interaction with soil type, climate, and crops grown. Much of the research on the beneficial effects of biochar has been conducted in temperate and tropical regions such as North America, East Asia, and Europe^1^. In these regions, soils are generally acidic, and the application of biochar has been shown to have a liming effect on soil pH^11,24^. However, relatively little is known about the impact of biochar on crop growth and performance in the forest-savanna transition zone of southwest Nigeria, where the soils are acidic. Therefore, the objective of this study was to assess how the addition of *Tithonia diversifolia* biochar affects soil physicochemical properties, leaf nutrient concentrations, and the growth of broccoli on severely degraded sandy loam soil in southwest Nigeria.

## Results

### Initial soil analysis

The physical and chemical properties of the soil used for the experiment are presented in Table 1. The soil was sandy loam in texture, acidic, and had high bulk density and low total porosity. The 0.96% soil organic carbon (OC), 0.08% total N, 3.85 mg kg^− 1^ available P, 0.11 cmol kg^− 1^ exchangeable K, 1.63 cmol kg^− 1^ exchangeable Ca and 0.32 cmol kg^− 1^ exchangeable Mg were all very low. Additionally, the cation exchange capacity (CEC) of 2.55 cmol kg^− 1^ and exchangeable acidity of 0.40 cmol kg^− 1^ were also very low, indicating poor soil fertility. Therefore, the soil is unlikely to sustain crop yields without the addition of external inputs.

### Physical and chemical properties of *Tithonia diversifolia* used for the experiment

The physical and chemical properties of the *Tithonia diversifolia* used for the experiment are shown in Table 2. The Biochar exhibited high values of electrical conductivity (EC), nitrogen (N), potassium (K), calcium (Ca) contents, and other micronutrients at the level required for the growth of broccoli. The application of Biochar in the short term is expected to benefit crops and soil. It is alkaline with a pH value of 7.89.

### Effects of *Tithonia diversifolia* Biochar on physical properties of soil

The effects of *Tithonia diversifolia* Biochar application on soil bulk density, total porosity, and moisture content are presented in Table 3. The application of Biochar resulted in lower bulk density across all treatments compared to the control. Additionally, Biochar application led to increased moisture content and porosity. As the level of Biochar application increased, soil bulk density decreased, while porosity and moisture content showed a corresponding increase compared to the control. Application of tithonia Biochar at 10, 20, 30, and 40 t ha^− 1^ decreased bulk density by 13%, 20%, 26%, and 33%, respectively, compared to the control. Application of tithonia Biochar at 10, 20, 30, and 40 t ha^− 1^ increased porosity by 20%, 32%, 41%, and 51%, respectively, compared to the control, while the same treatments increased moisture content by 24%, 44%, 59%, and 75%, respectively, compared to the control.

**Table 1:** Physical and chemical properties of soil (0–15 cm depth) used for the experiment.

Property	Value
Sand (%)	77.1
Silt (%)	7.3
Clay (%)	15.6
Textural class	Sandy loam
Bulk density (Mg m^− 3^)	1.63
Total porosity (%)	38.5
pH (water)	5.62
Organic carbon (%)	0.96
Total N (%)	0.08
Available P (mg kg^− 1^)	3.85
Exchangeable K (cmol kg^− 1^)	0.11
Exchangeable Ca (cmol kg^− 1^)	1.63
Exchangeable Mg (cmol kg^− 1^)	0.32
Exchangeable Na (cmol kg^− 1^)	0.09
Exchangeable acidity (cmol kg^− 1^)	0.40
Cation exchange capacity (cmol kg^− 1^)	2.55

**Table 2:** Chemical composition of Biochar (*Tithonia diversifolia*) used in the experiment

Property	Value
Electrical conductivity (dS m^− 1^)	1.45
Surface area (m^2^ g^− 1^)	11.8
pH (water)	7.89
Cation exchange capacity (cmol kg^− 1^)	25.68
Organic carbon (%)	22.32
Nitrogen (%)	1.36
C/N	16.41
Phosphorus (%)	0.43
Potassium (%)	2.19
Calcium (%)	1.82
Magnesium (%)	0.70
Sodium (mg kg^− 1^)	4444
Sulphur (mg kg^− 1^)	3635
Copper (mg kg^− 1^)	73.33
Manganese (mg kg^− 1^)	58.60
Iron (mg kg^− 1^)	24.44
Cation exchange capacity (cmol kg^− 1^)	25.68

**Table 3:** Effects of *Tithonia diversifolia* Biochar on physical properties of soil (0–10 cm depth).

Treatment	Bulk density (Mg m^− 3^)	Total porosity (%)	Moisture content (%)
0 t ha^− 1^ (control)	1.61a	39.2e	8.7e
10 t ha^− 1^ tithonia biochar	1.40b	47.2d	10.8d
20 t ha^− 1^ tithonia biochar	1.28c	51.7c	12.5c
30 t ha^− 1^ tithonia biochar	1.19d	55.1b	13.8b
40 t ha^− 1^ tithonia biochar	1.08e	59.2a	15.2a

Means followed by the same letter on a column are not significantly different at *p* = 0.05 according to Duncan’s multiple range test (DMRT)

**Table 4:** Effects of *Tithonia diversifolia* Biochar on soil chemical properties (0–15 cm depth)

Treatment	pH (H_2_O)	OC (%)	Total N (%)	Avail. P (mg kg^− 1^)	Exch. K (cmol kg^− 1^)	Exch. Ca (cmol kg^− 1^)	Exch. Mg (cmol kg^− 1^)
0 t ha^− 1^ (control)	5.58e	0.89e	0.07e	3.73e	0.10e	1.42e	0.42e
10 t ha^− 1^ tithonia biochar	5.89d	1.07d	0.09d	4.58d	0.15d	1.65d	0.65d
20 t ha^− 1^ tithonia biochar	6.24c	1.35c	0.12c	6.45c	0.19c	1.93c	0.83c
30 t ha^− 1^ tithonia biochar	6.56b	1.63b	0.15b	8.32b	0.22b	2.16b	1.02b
40 t ha^− 1^ tithonia biochar	6.88a	2.04a	0.19a	10.23a	0.25a	2.47a	1.86a

Means followed by the same letter on a column are not significantly different at *p* = 0.05 according to Duncan’s multiple range test (DMRT)

**Table 5:** Effects of *Tithonia diversifolia* Biochar on leaf nutrient concentration of broccoli.

Treatment	N (%)	P (mg kg^− 1^)	K (mg kg^− 1^)	Ca (mg kg^− 1^)	Mg (mg kg^− 1^)
0 t ha^− 1^ (control)	2.10d	0.29e	1.96e	1.76e	0.68e
10 t ha^− 1^ tithonia biochar	2.80c	0.42d	2.84d	1.98d	0.79d
20 t ha^− 1^ tithonia biochar	4.02a	0.68a	3.98a	2.85a	1.28a
30 t ha^− 1^ tithonia biochar	3.50b	0.56b	3.42b	2.54b	0.96b
40 t ha^− 1^ tithonia biochar	3.29b	0.48c	3.05c	2.18c	0.85c

Means followed by the same letter on a column are not significantly different at *p* = 0.05 according to Duncan’s multiple range test (DMRT)

**Table 6:** Effects of *Tithonia diversifolia* Biochar on the growth parameters of broccoli at 90 days after sowing.

Treatment	Plant height (cm)	Number of leaves per plant	Leaf area (cm^2^)	Stem girth (cm)	Fresh weight of broccoli biomass (g)
0 t ha^− 1^ (control)	13.3d	3.2d	385.32c	8.33c	147.24d
10 t ha^− 1^ tithonia biochar	18.1c	4.4c	450.45b	9.89b	162.15c
20 t ha^− 1^ tithonia biochar	26.1a	5.8a	495.86a	10.78a	184.61a
30 t ha^− 1^ tithonia biochar	20.9b	4.9b	458.79b	9.61b	169.52b
40 t ha^− 1^ tithonia biochar	20.6b	4.8b	449.96b	9.58b	165.63b

Means followed by the same letter on a column are not significantly different at *p* = 0.05 according to Duncan’s multiple range test (DMRT).

### Effects of *Tithonia diversifolia* Biochar on soil chemical properties

 Application of *Tithonia diversifolia* influenced soil chemical properties (Table 4). The application of tithonia Biochar at rates of 10, 20, 30, and 140 t ha^-1^ resulted in increased soil pH, OC, TN, P, K, Ca, and Mg. As the amount of tithonia Biochar increased, there was a corresponding increase in soil pH, OC, TN, P, K, Ca, and Mg. Particularly, applying tithonia Biochar at rates of 10, 20, 30, and 40 t ha^-1^ led to the higher soil pH, OC, TN, P, K, Ca, and Mg compared to the control. Application of tithonia Biochar at 10 t ha^-1^ increased soil pH, OC, TN, P, K, Ca, and Mg by 6%, 20%, 29%, 23%, 50%, 16%, and 41%, respectively, compared to the control. Application of tithonia Biochar at 20 t ha^-1^ increased soil pH, OC, TN, P, K, Ca, and Mg by 12%, 52%, 71%, 73%, 90%, 36%, and 117%, respectively, compared to the control. Application of tithonia Biochar at 30 t ha^-1^ increased soil pH, OC, TN, P, K, Ca, and Mg by 18%, 83%, 114%, 123%, 120%, 52%, and 159%, respectively, compared to the control. Application of tithonia Biochar at 40 t ha^-1^ increased soil pH, OC, TN, P, K, Ca, and Mg by 23%, 129%, 171%, 174%, 150%, 74%, and 197%, respectively, compared to the control.

### Effects of *Tithonia diversifolia* Biochar on leaf nutrient concentration of broccoli

 Table 5 presents the effects of *Tithonia diversifolia* Biochar on the leaf nutrient concentrations of broccoli at the flowering stage, four months after sowing. The application of tithonia Biochar significantly influenced the leaf nutrient concentrations of broccoli. Specifically, tithonia Biochar application significantly (*p* = 0.05) increased the concentrations of nitrogen (N), phosphorus (P), potassium (K), calcium (Ca), and magnesium (Mg) in the leaves, with these concentrations increasing in response to higher rates of tithonia Biochar application compared to the control group. The concentrations of N, P, K, Ca, and Mg in the broccoli leaves increased with tithonia Biochar application rates up to 20 t ha^-1^. However, beyond this rate, the concentration of N, P, K, Ca, and Mg in the leaves was decreased by the tithonia Biochar application. The application of tithonia Biochar at a rate of 20 t ha^-1^ resulted in the highest leaf concentrations of N, P, K, Ca, and Mg compared to other treatments. The application of tithonia Biochar at a rate of 20 t ha^-1^ increased leaf concentrations of N, P, K, Ca, and Mg by 99%, 134%, 103%, 62%, and 88%, respectively, compared to other treatments.

### Effects of *Tithonia diversifolia* Biochar on the growth parameters of broccoli

The effects of *Tithonia diversifolia* Biochar on the growth parameters of broccoli, including plant height, number of leaves, leaf area, stem girth, and fresh weight of broccoli biomass, observed 90 days after sowing, are presented in Table 6. The application of tithonia Biochar significantly influenced the growth of broccoli. It was noted that the plant height, number of leaves, leaf area, stem girth, and fresh weight of broccoli biomass increased significantly (*p* = 0.05) with increasing tithonia Biochar application rates, from 0 to 40 t ha^− 1^. Among the various application rates, the rate of 20 t ha^− 1^ was found to be the most effective, resulting in the highest plant height, number of leaves, leaf area, stem girth, and fresh weight of broccoli biomass (Table 6). However, application rates above 20 t ha^− 1^, such as 30 and 40 t ha^− 1^, tended to decrease these growth parameters, indicating a diminishing return in production. This suggests that while tithonia Biochar can enhance the growth of broccoli up to a certain threshold, excessive application may have adverse effects. The application of tithonia Biochar at rates of 10, 20, 30, and 40 t ha^− 1^ increased fresh broccoli biomass by 13%, 38%, 26%, and 23%, respectively, compared to the control.

## Discussion

The soil’s low nutrient status is due to its sandy loam texture, which limits nutrient retention, coupled with high acidity, high bulk density, and low porosity, all of which hinder root development and nutrient uptake. Critical fertility indicators, including organic carbon, nitrogen, phosphorus, and essential cations (K, Ca, and Mg), fall below recommended critical levels of 3.0% OM, 0.20% N, 10.0 mg kg^− 1^ available P, 0.16–0.20 cmol kg^− 1^ exchangeable K, 2.0 cmol kg^− 1^ exchangeable Ca, and 0.40 cmol kg^− 1^ exchangeable Mg^25^, while low cation exchange capacity (CEC) further reduces the soil’s ability to retain nutrients. As a result, the soil requires external inputs to support sustainable crop production.

The application of tithonia biochar at varying levels reduced soil bulk density while increasing moisture content and porosity. These improvements can be attributed to enhanced soil organic matter (SOM) resulting from the decomposition of tithonia biochar by soil microorganisms, consistent with previous findings from laboratory incubation studies using organic amendments^26,27^. The reductions in bulk density and increases in porosity and moisture content likely created a more favorable soil environment for root growth and microbial respiration^28^. This study confirmed that biochar application significantly decreased the bulk density of the sandy loam soil analyzed (Table 3), aligning with prior research on both fine-textured soils^29^ and coarse-textured soils^30^. The reduction in bulk density can be attributed to biochar’s inherently low bulk density (< 0.6 Mg m⁻³) compared to the field soil (~ 1.2 Mg m⁻³), which creates a dilution or mixing effect^22^. Additionally, biochar interacts with soil particles, promoting aggregation and increasing porosity^31^. Lim et al.^30^ further noted that biochar can modify soil particle arrangement, resulting in greater external porosity. Overall, the reduction in bulk density following biochar application highlights its potential to enhance soil structure, stability, and overall biophysical conditions.

The application of tithonia biochar resulted in increased concentrations of soil pH, organic carbon (OC), phosphorus (P), potassium (K), calcium (Ca), and magnesium (Mg). The rise in soil pH observed at different biochar application rates can be attributed to the increased availability of organic matter and high concentrations of alkali metals and exchangeable basic cations (Ca, Mg, K, and Na) in the biochar’s ash fractions, which act as liming agents in acidic soils. The enhanced levels of soil OC and nutrients in biochar-amended plots, compared to the control, can be explained by several mechanisms: the addition of nutrients from the biochar, reduced nutrient leaching, improved nutrient retention, modified soil microbial dynamics, and increased decomposition of organic materials in the soil, as reported in previous studies^23,32^. Major et al.^33^ demonstrated that biochar has the ability to retain nutrients contained in water solutions held in its micropores by capillary forces. According to the authors, biochar particles are assumed to behave like clay, holding large amounts of immobile water even at increased matric potentials. Consequently, nutrients dissolved in this immobile water remain near the soil surface, making them available for plants^33^. Additionally, Major et al.^33^ found that biochar reduces the leaching of soil nutrients through the adsorption of cations and anions to its surfaces. The mechanisms responsible for the increased availability of plant nutrients in biochar-amended plots include the rise in soil pH (in acidic soils), high OC content, stability, nutrient retention (due to increased cation exchange capacity and surface area), and the direct release of nutrients from biochar surfaces^34–36^.

The application of tithonia biochar at different rates resulted in the highest broccoli leaf concentrations of N, P, K, Ca, and Mg compared to the control due to biochar’s ability to enhance soil nutrient retention, improve microbial activity, and increase nutrient availability. Additionally, biochar’s porous structure and high cation exchange capacity promote efficient nutrient uptake by plants. The application of tithonia biochar at 20 t ha^− 1^ resulted in the highest leaf concentrations of N, P, K, Ca, and Mg because this rate provided an optimal balance of nutrients, enhancing soil fertility and nutrient availability without causing nutrient immobilization or antagonistic effects. However, applying biochar at rates above 20 t ha⁻¹, such as 30 and 40 t ha⁻¹, likely caused nutrient immobilization due to high organic matter, reducing nutrient availability and uptake efficiency, which led to lower leaf concentrations of these essential nutrients. Excessive biochar can also alter soil pH and microbial activity, further impacting nutrient availability and plant absorption. The application of tithonia biochar at 20 t ha⁻¹ provides optimal soil conditions, enhancing nutrient availability and uptake by broccoli plants, leading to higher leaf concentrations of N, P, K, Ca, and Mg. Higher application rates (30 and 40 t ha⁻¹) may result in nutrient imbalances or soil compaction, which can adversely affect root growth and nutrient absorption, thus lowering leaf nutrient concentrations.

The application of tithonia biochar at different rates enhances the growth parameters of broccoli due to its ability to improve soil fertility by increasing nutrient availability, water retention, and soil pH. This leads to better nutrient uptake by the plants, resulting in increased plant height, number of leaves, leaf area, stem girth, and fresh weight of the broccoli biomass compared to the control. The application of tithonia biochar at a rate of 20 t ha^− 1^ resulted in the highest growth parameters of broccoli due to its optimal enhancement of soil fertility, and nutrient availability. Rates above 20 t ha^− 1^, such as 30 and 40 t ha^− 1^, likely led to reduced plant growth parameters because of potential nutrient imbalances or toxicity, which can hinder plant development.

## Conclusion

The application of tithonia biochar significantly improved the physical and chemical properties of the soil, including bulk density, porosity, moisture content, pH, organic carbon (OC), total nitrogen (TN), phosphorus (P), potassium (K), calcium (Ca), and magnesium (Mg). These improvements in soil quality translated into better nutrient accumulation in broccoli plants, as well as enhanced growth metrics such as plant height, number of leaves, leaf area, stem girth, and fresh biomass weight. Among the different application rates, 20 t ha^− 1^ was the most effective in improving leaf nutrient concentrations and growth parameters, followed by 30 and 40 t ha^− 1^, in that order. The primary mechanisms behind these improvements include increased organic matter content, enhanced water retention, elevated soil pH, and improved availability of both macro and micronutrients to plants, leading to greater nutrient accumulation in the broccoli plants.

## Materials and methods

### Description of the study area

**Fig. 1:**
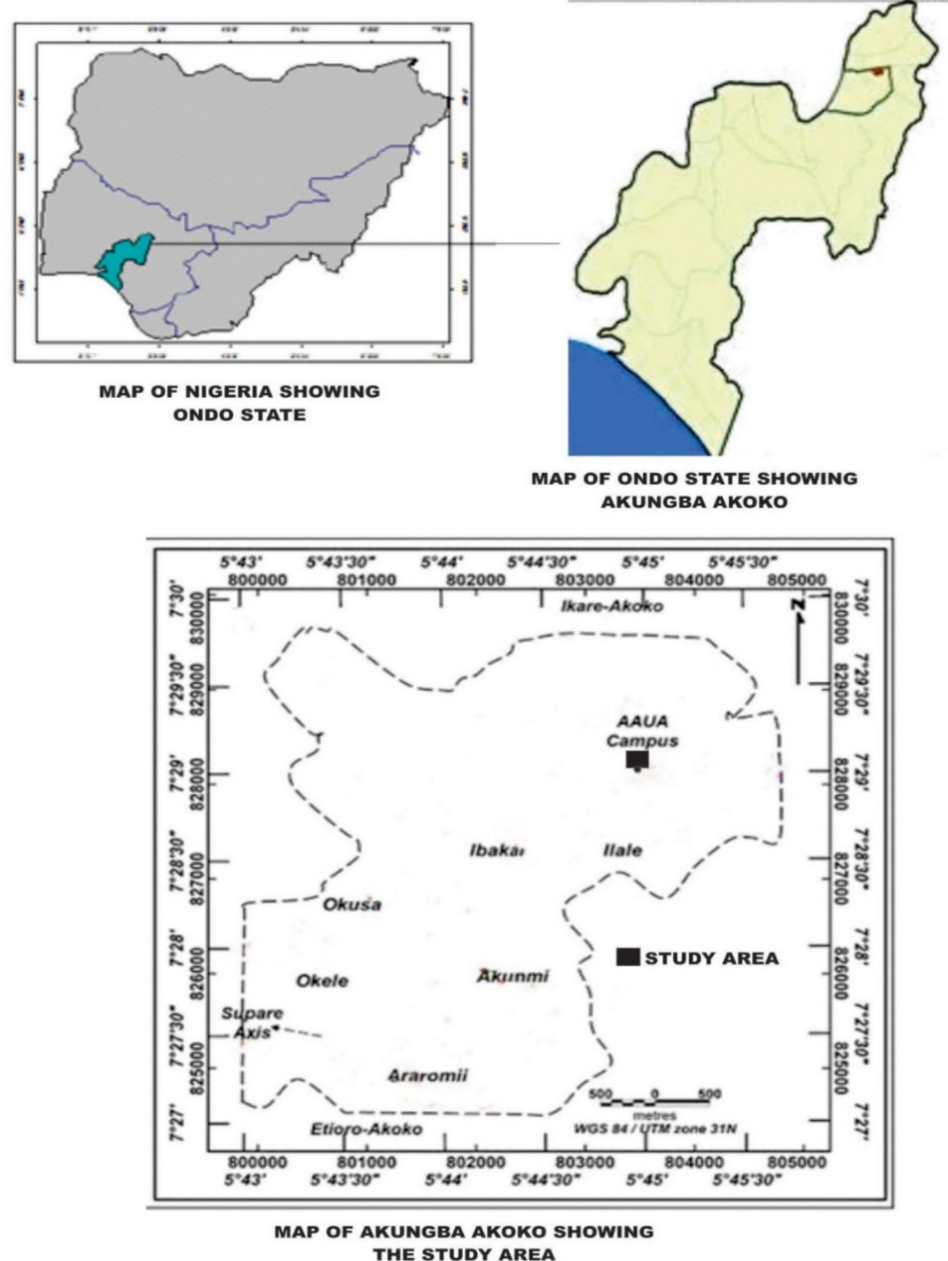
Showing Map of the study area.

 The experiment was carried out at the Teaching and Research Farm, Adekunle Ajasin University, Akungba-Akoko, Ondo State, Nigeria (Fig. 1). The site is located at Latitudes 7° 28” 9.15’ to 7° 29” 15.18’ North of the equator and longitudes 5° 44” 15.96’ to 5° 46” 14.78’ East of the Greenwich Meridian^37^, with altitudes ranging from 317 to 352 m above sea level. Soils of the area were formed predominantly from Precambrian basement complex rocks such as gray gneiss, quartzo-feldspathic gneiss, charnockite, granite gneiss, and porphyritic gneiss^38^, which form parts of the African crystalline shield^39^. Rainfall is bimodal, with mean annual rainfall ranging between 1200 and 1400 mm, with most occurring from March to July and from mid-August to November. The mean annual air temperature is between 21 and 32 °C with relative humidity varies from 75 to 95%. The natural rainforest vegetation that previously characterized the study area gradually receded to derived savannah due to human activities.

### Biochar preparation

 Fresh sunflowers (*Tithonia diversifolia*) plants were harvested from the field, and the stems and leaves are collected. The biomass was then air-dried for 2 weeks to reduce its moisture content, which is crucial for efficient pyrolysis. The dried biomass was chopped into smaller pieces to increase the surface area, facilitating uniform pyrolysis. The *Tithonia diversifolia* was burnt under low oxygen (pyrolysis) in a homemade biochar kiln, constructed from a repurposed 200-liter (55-gallon) metal drum and operating at a temperature of 350 °C. After pyrolysis, the biochar was allowed to cool in an inert atmosphere for approximately 4–6 h to prevent oxidation. After cooling, the biochar was ground to a uniform particle size (2–3 mm) prior to experimental use.

### Chemical analysis of ***Tithonia diversifolia*** used for the experiment

Small quantities of approximately 5 g subsamples of the *Tithonia diversifolia* used in the experiments were analyzed to determine its nutrient composition. The samples were air-dried and crushed to pass through a 2-mm sieve before analysis. The samples were analyzed for pH, electrical conductivity (EC), specific surface area, organic carbon (OC), total nitrogen (TN), available P, exchangeable K, exchangeable Ca, and exchangeable Mg, and S as well as the concentrations of trace elements such as manganese (Mn), iron (Fe), copper (Cu), zinc (Zn), and sodium (Na) as described by Tel and Hagarty^40^. Cation exchange capacity (CEC) was also determined. The pH and EC of the biochar were determined in a 1:10 (biochar: distilled water) ratio^41^. The specific surface area was determined using the Brunauer-Emmett-Teller method^42^. The percentage of OC was determined by the Walkley and Black procedure using the dichromate wet oxidation method, and total N was determined by micro-Kjeldahl digestion, followed by distillation and titration. The determination of P, K, Ca and Mg was performed using the wet digestion method based on 25-5-5 mL of HNO_3_-H_2_SO_4_-HClO_4_ acids. Phosphorus was measured colorimetrically by the molybdate blue method in an autoanalyzer, K was measured by flame photometry, and Ca and Mg were measured by an atomic absorption spectrophotometer. A vario MACRO cube elemental analyzer was used for the quantification of sulfur content of the *Tithonia diversifolia* sample. To determine the concentrations of trace elements, such as Mn, Fe, Cu, Zn, and Na, sample of *Tithonia diversifolia* with known quantity was incinerated at 760 °C in a muffle furnace. The resulting ash was treated with HCl, diluted with deionized water, and then analyzed for trace element concentrations. Mn, Fe, Cu, and Zn levels were determined in *Tithonia diversifolia* sample through the use of an atomic absorption spectrophotometer, while the concentration of Na was measured using a flame photometer. Cation exchange capacity (CEC) was determined using the ammonium acetate method at pH 7.

### Potting, treatment and crop establishment

The experiment was conducted inside a screen house at the Faculty of Agriculture, Adekunle Ajasin University, Akungba-Akoko, Ondo State, between May and October, 2022. The experiment was repeated twice. Each experiment was terminated after 90 days after sowing. Fifteen (15) polythene bags, each measuring 30 × 30 × 30 cm, were filled with 10 kg air-dried soil and thoroughly mixed with five application rates of *Tithonia diversifolia* biochar (0, 10, 20, 30 and 40 t ha ^− 1^). That is quantities of 0 g, 50 g, 100 g, 150 g, and 200 g of ***Tithonia diversifolia*** biochar were mixed into 10 kg of soil in the grow bags, corresponding to the equivalent rates of 0, 10, 20, 30, and 40 t ha^− 1^ of ***Tithonia diversifolia*** biochar, respectively. The experiment was laid out in a completely randomized design (CRD) with three replications, 12 h photoperiod, day 25 ± 2 °C and night temperature 17 ± 3 °C. Each polythene bag was maintained at 70% field moisture capacity throughout the course of the experiment. The bags were incubated in the screen house for four (4) weeks before sowing. After the incubation period, five (5) healthy broccoli (*Brassica oleracea*) seeds (hybrid FI ISABELA), obtained from University of Ilorin, were sown in each bag. After germination, three (3) plants were maintained in each polythene bag. Each pot was watered daily with tap water till harvest.

### Determination of growth parameters of broccoli (*Brassica olericea*)

 Three plants were selected per pot for data collection. The growth parameters determined were plant height, number of leaves, leaf area, stem girth, and fresh weight of broccoli biomass. The plant height and number of leaves were determined at 90 days after sowing. The plant height was measured with a meter rule, number of leaves was determined by counting. The leaf area was determined by graphical method and fresh weight of broccoli biomass was determined by using a sensitive weighing balance.

### Soil analysis

 Soil samples were collected with an auger before the commencement of the experiment and at a depth of 0–15 cm. Additional samples were taken from each polythene bag at the end of the experiment for analysis. The soil samples collected were bulked, air-dried and sieved using a 2-mm sieve for routine physical and chemical analysis, as described by Carter and Gregorich^43^. Particle-size analysis was performed using the hydrometer method. The textural class was determined using a textural triangle. Bulk density and moisture content were determined using a coring tube (3 cm diameter, 10 cm height), while moisture content was specifically measured using the gravimetric method. Total porosity was then calculated from the bulk density value of 2.65 Mg m^− 3^. The soil pH was determined in a soil/water (1:2) suspension using a digital electronic pH meter. Soil organic carbon was determined by the Walkley and Black procedure by wet oxidation using chromic acid digestion. Total N was determined using micro-Kjeldahl digestion and distillation techniques, and available P was determined by Bray-1 extraction followed by molybdenum blue colorimetry. Exchangeable K, Ca, Mg, and Na were extracted with a 1 N ammonium acetate (NH_4_OA_C_) solution (pH 7). Thereafter, exchangeable K and Na were analyzed with a flame photometer, while exchangeable Ca and Mg were determined with an atomic absorption spectrophotometer. Cation exchange capacity (CEC) was measured using the ammonium acetate method at pH 7, and exchangeable acidity was determined by titration with NaOH after extraction with 1 N KCl.

### Leaf analysis of broccoli plant

 Leaf samples were collected from each polythene bag at the end of the experiment to determine their nutrient composition. Fully expanded mature leaves were specifically selected for analysis. The leaves were washed thoroughly with distilled water. These plant samples were air-dried for 3 days and then oven dried at 70ºC for 48 h until a constant weight was achieved. Using plant grinder, the dried plant samples were ground to 40 mesh. Leaf N was determined by micro-Kjeldahl digestion method. Samples were dry ashed at 500 °C for 6 h in a furnace and extracted using nitric-perchloric-sulfuric acid mixture for determination of P, K, Ca, and Mg. Leaf phosphorus was determined colorimetrically by the vanadomolybdate method. Potassium was determined using a flame photometer, and Ca and Mg were determined by the ethylene diamine tetra acetic acid (EDTA) titration method^44^.

### Statistical analysis

 The collected data were subjected to one-way analysis of variance (ANOVA) using the Statistical Analysis System (SAS, Version 9.4)^45^. The treatment means were separated using Duncan’s multiple range test (DMRT) at *p* = 0.05 probability level. Since there was no significant difference between the two experiments, the data were pooled for analysis.

## Data Availability

All datasets generated and/or analysed during the current study are included in this article.
